# External validation of a multivariable prediction model for positive resection margins in breast-conserving surgery

**DOI:** 10.1186/s13104-025-07103-8

**Published:** 2025-01-27

**Authors:** Irina Palimaru Manhoobi, Julia Ellbrant, Pär-Ola Bendahl, Søren Redsted, Anne Bodilsen, Trine Tramm, Peer Christiansen, Lisa Rydén

**Affiliations:** 1 Department of Radiology, Aarhus University Hospital, Aarhus, Denmark; 2 Department of Surgery, Department of Clinical Sciences, Division of Surgery, Skåne University Hospital, Lund University, Lund, Sweden; 3 Department of Clinical Sciences, Division of Oncology, Lund University, Lund, Sweden; 4 Department of Abdominal Surgery, Aarhus University Hospital, Aarhus, Denmark; 5 Department of Pathology, Aarhus University Hospital, Aarhus, Denmark; 6 Department of Plastic and Breast Surgery, Aarhus University Hospital, Aarhus, Denmark; 7 Department of Clinical Medicine, Aarhus University, Aarhus, Denmark; 8 Department of Radiology, Palle Juul Jensens Boulevard 99, Aarhus N, 8200 Denmark

**Keywords:** Breast-conserving surgery, Prediction model, External validation, Positive resection margins, Invasive breast cancer

## Abstract

**Objectives:**

Positive resection margins after breast-conserving surgery (BCS) most often demands a repeat surgery. To preoperatively identify patients at risk of positive margins, a multivariable model has been developed that predicts positive margins after BCS with a high accuracy. This study aimed to externally validate this prediction model to explore its generalizability and assess if additional preoperatively available variables can further improve its predictive accuracy. The validation cohort included 225 patients with invasive breast cancer who underwent BCS at Aarhus University Hospital, Aarhus, Denmark during 2020–2022. Receiver operating characteristic (ROC) and calibration analysis were used to validate the prediction model. Univariable logistic regression was used to evaluate if additional variables available in the validation cohort were associated with positive margins and backward elimination to explore if these variables could further improve the model´s predictive accuracy.

**Results:**

The AUC of the model was 0.60 (95% CI: 0.50–0.70) indicating a lower discriminative capacity in the external cohort. We found weak evidence for an association between increased preoperative breast density on mammography and positive resection margins after BCS (*p* = 0.027), but the AUC of the model did not improve, when mammographic breast density was included as an additional variable in the model.

**Supplementary Information:**

The online version contains supplementary material available at 10.1186/s13104-025-07103-8.

## Introduction

Breast cancer is the most common cancer among women worldwide [1]. The standard treatment of invasive breast cancer is breast-conserving surgery (BCS) followed by radiotherapy [2, 3]. It is well established that women undergoing BCS may require additional surgeries, due to positive resection margins with invasive or non-invasive cancer in the final histopathology with reported prevalence from 5% up to 42% [4, 5]. The high variability in reported prevalences of repeat surgeries is due to use of different inclusion criteria like invasive cancer only or combined invasive and in situ breast cancer [6–14], and the use of different definitions for margin positivity [15].

Repeat surgery increases risk of anxiety for the patients [16], impairs the cosmetic outcome [17], prolongs the time to systemic treatment [4, 18], and increases health care costs [19].

To identify patients at high risk of positive margins following BCS, various prediction models have been developed [8–11, 14], but only few of these have been externally validated [20–22].

In 2021, Ellbrant et al. published a multivariable model that predicted positive margins after BCS with an area under the ROC curve (AUC) of 0.80 using 7 preoperative available variables:

(1) Invasive lobular cancer, (2) ductal carcinoma in situ (DCIS), (3) tumor size, (4) no visible tumor on mammography, (5) mammographic microcalcifications, (6) distance to the nipple-areola complex (NAC) less than 5 cm, and (7) planned oncoplastic surgery [10]. The generalizability of this model to a non-Swedish setting has not yet been investigated. Other studies have also found an association between high mammographic breast density and positive margins after BCS [5, 8, 23].

The primary aim of the present study was to externally validate the prediction model.

A secondary aim was to explore if additional variables, such as high mammographic breast density predicts positive margins after BCS and can further improve the accuracy of the model in the validation cohort.

## Methods

### Validation cohort

This observational cohort study included women in the validation cohort from a previous randomized controlled trial [5] with invasive breast cancer confirmed by core-needle biopsy, age years ≥ 18 that underwent BCS between September 2020 and January 2022 at the Department of Plastic and Breast Surgery, Aarhus University Hospital, Aarhus, Denmark. Two of the patients with DCIS without invasive cancer in the core-needle biopsy were included unintentionally in the previous randomized trial. We decided to include these two patients in this present study, as DCIS was not an exclusion criterion in the development cohort. Patients treated with a planned mastectomy, or with neoadjuvant chemotherapy (NACT) were excluded.

A positive histopathological resection margin was defined as a 0 mm margin for invasive cancer, and < 2 mm for DCIS [24–26].

### Statistical analysis

The available data from the original study was extracted [5, 27] and used for the external validation. Hence, no formal sample size calculation was performed. Associations between categorical predictors of the model and positive resection margins after BCS, were analyzed using univariable logistic regression analysis. Comparison of patient and tumor characteristics between the cohorts was performed using independent samples t-test for the continuous variables and Pearson’s chi-squared test or Fisher´s exact test for the categorical variables. Linear regression was used to test for trend for categorical variables with more than two ordered categories.

One of the predictors, the dichotomized distance to the nipple-areola complex, had 34 missing values in the validation cohort (Table 1). To be able to include also patients with incomplete data, ten complete datasets were created using a logistic regression model [28] and the missing at random (MAR) assumption was fulfilled conditional for the imputation model. However, DCIS was excluded from the imputation model due to low prevalence (2/225) and tumor visibility on mammography because of collinearity. To strengthen the support for the (MAR) assumption, mammographic breast density was added to the logistic imputation model as 70% (24/34) of the patients with missing values had increased mammographic breast density.

**Table 1:** Patient characteristics of the 225 study patients in the validation cohort

	Total (%) *n* = 225	Clear margins (%) *n* = 184	Positive margins (%) *n* = 41	Odds Ratio (95% CI)	*P* value
**Demographic characteristics**					
Age, years [mean (min-max)] < 50 50–59 60–69 ≥ 70	65 (32–90) 13 (5.8) 51 (22.7) 93 (41.3) 68 (30.2)	65 (32–90) 11 (6.0) 37 (20.1) 76 (41.3) 60 (32.6)	63 (45–86) 2 (4.9) 14 (34.1) 17 (41.5) 8 (19.5)	0.98 (0.95, 1.01) 1.36 (0.25, 7.30) 2.84 (1.09, 7.41) 1.68 (0.68, 4.15) 1.00 (reference)	0.288^a^ 0.098^b^
**Radiological features**					
Visibility on mammography Visible Not visible	196 (87.1) 29 (12.9)	164 (89.1) 20 (10.9)	32 (78.1) 9 (22.0)	1.00 (reference) 2.31 (0.96, 5.52)	0.061^a^
Mammographic tumour size, mm [median (min-max)] ≤ 20 (T1) 21–50 (T2) Not visible Not measurable*	14 (4–77) 161 (71.6) 30 (13.3) 29 (12.9) 5 (2.2)	13.5 (4–49) 137 (74.4) 23 (12.5) 20 (10.9) 4 (2.2)	14 (4–77) 24 (58.5) 7 (17.1) 9 (22.0) 1 (2.4)	1.019 (0.979, 1.061) 1.00 (reference) 1.74 (0.67, 4.50) 2.57 (1.05, 6.31) 1.43 (0.15, 13.32)	0.358^a^ 0.255^a^
Mammographic calcifications Yes No	27 (12.0) 198 (88.0)	20 (10.9) 164 (89.1)	7 (17.1) 34 (82.9)	1.69 (0.66, 4.31) 1.00 (reference)	0.273^a^
Mammographic distance NAC (cm) < 5 ≥ 5 Missing**	45 (20.0) 146 (64.9) 34 (15.1)	34 (18.5) 126 (68.5) 24 (13.0)	11 (26.8) 20 (48.8) 10 (24.4)	2.04 (0.89, 4.66) 1.00 (reference)	0.092^a^
Ultrasonographic tumour size, mm [mean (min-max)] ≤ 20 (T1) 21–50 (T2) Not visible	13 (3–40) 190 (84.4) 32 (14.2) 3 (1.33)	13 (4–40) 160 (87.0) 23 (12.5) 1 (0.5)	14 (3–37) 30 (73.2) 9 (22.0) 2 (4.9)	1.027 (0.975, 1.081) 1.00 (reference) 2.09 (0.88, 4.95) 10.67 (0.94, 121.39)	0.317^a^ 0.095^a^
**Clinical-pathological findings**					
Palpability Palpable Non-palpable	107 (47.6) 118 (52.4)	85 (46.2) 99 (53.8)	22 (53.7) 19 (46.3)	1.00 (reference) 0.74 (0.38, 1.46)	0.388^a^
Tumour location Superior medial quadrant Superior lateral quadrant Inferior lateral quadrant Inferior medial quadrant Retromammillary	41 (18.2) 134 (59.6) 23 (10.2) 15 (6.7) 12 (5.3)	29 (15.8) 117 (63.6) 17 (9.2) 11 (6.0) 10 (5.4)	12 (29.3) 17 (41.5) 6 (14.6) 4 (9.7) 2 (4.9)	1.00 (reference) 0.35 (0.15, 0.82) 0.85 (0.27, 2.69) 0.88 (0.23, 3.31) 0.48 (0.09, 2.54)	0.051^b^
Core-needle biopsy Lobular cancer Yes No	32 (14.2) 193 (85.8)	24 (13.0) 160 (87.0)	8 (19.5) 33 (80.5)	1.62 (0.67, 3.91) 1.00 (reference)	0.287^a^
Core-needle biopsy: DCIS Yes No	2 (0.9) 223 (99.1)	1 (0.5) 183 (99.5)	1 (2.4) 40 (97.6)	4.58 (0.28, 74.70) 1.00 (reference)	0.332^c^
**Type of surgery**					
Partial mastectomy Oncoplastic partial mastectomy	214 (95.1) 11 (4.9)	177 (96.2) 7 (3.8)	37 (90.2) 4 (9.8)	1.00 (reference) 2.73 (0.76, 9.82)	0.123^a^
**Additional clinical characteristics**					
Menopausal status Premenopausal Postmenopausal	31 (13.8) 194 (86.2)	22 (12.0) 162 (88.0)	9 (22.0) 32 (78.0)	2.07 (0.87, 4.91) 1.00 (reference)	0.098^a^
**Additional radiological features**					
Breast density mammography A B C D	39 (17.3) 111 (49.3) 66 (29.3) 9 (4.0)	34 (18.5) 93 (50.5) 53 (28.8) 4 (2.2)	5 (12.2) 18 (43.9) 13 (31.7) 5 (12.2)	1.00 (reference) 1.31 (0.45, 3.82) 1.67 (0.55, 5.10) 8.50 (1.69, 42.76)	**0.027** ^**b**^
Breast magnetic resonance imaging Yes No	65 (28.9) 160 (71.1)	48 (26.1) 136 (73.9)	17 (41.5) 24 (58.5)	2.01 (0.99, 4.05) 1.00 (reference)	0.052^a^

**Abbreviations:** n, number of patients; CI, Confidence Interval; NAC, Nipple-aerola-complex; DCIS, ductal carcinoma in situ, a) Univariable logistic regression analysis, b) Chi-square test for trend and c) Fischer´s exact test. *In 5 cases, the tumour was identified on mammography but the tumour margins where not clearly visible, with no measurable tumour. **Missing due to no visible tumour in 29 cases and no measurable tumour in 5 cases

External validation of the model [10] was performed by comparison of the predicted probability of positive resection margins for each patient according to the model and the histopathological margin status. Discrimination between positive and clear margins was quantified by AUC and the calibration illustrated using a Hosmer-Lemeshow graph.

Performance measures summarizing model discrimination (AUC) and calibration (calibration slope and intercept) were calculated as averages over the ten imputations. Backward elimination logistic regression analysis was used to explore if any of the additional imaging variables could be used to improve the model´s AUC.

The variables in the original prediction model were not subject to selection or reweighting.

Stata 17 (StataCorp, 2021, College Station, Texas, USA) was used for all the statistical analyses.

## Results

### Validation cohort

The validation cohort included 225 women with invasive breast cancer who underwent BCS. The median age was 65 years and the median tumor size on mammography 14 mm (Table 1). The support for associations between the predicting variables of the model and positive resection margins after BCS was in general low in the validation cohort (Table 1). A non-linear trend was observed for age with the highest odds of positive resection margins in the age category 50–59 years (Table 1), OR = 2.84 (95% CI: 1.09; 7.41) versus the chosen reference group ≥ 70 years. Furthermore, weak evidence for an association between increased preoperative breast density on mammography and positive resection margins after BCS (*p* = 0.027) was observed (Table 1).

### Comparison with the development cohort

The proportion of patients with positive resection margins after BCS in the external validation cohort was 18.2% (41/225), and 18.4% (41/223) when calculated for invasive cancers only (Table 1). The corresponding proportion of patients with positive margins in the development cohort was 17.8% (77/432), (Additional Figs. 1) and 13.6% (49/361), when calculated for invasive cancers only [10]. The cohorts were comparable regarding three out of the seven predictors in the model: Mammographic tumor size, distance from NAC, and percentage of patients with lobular cancer (Additional Table 1). However, the external validation cohort had a lower percentage of patients with pure DCIS (0.9% vs. 11.1%, *p* < 0.001); lower percentage of patients with microcalcifications on mammography (12.0% vs. 26.6%, *p* < 0.001); lower percentage of oncoplastic surgeries (4.9% vs. 28.5%, *p* < 0.001), and a higher percentage of patients with no visible tumor on preoperative mammography (12.9% *v*. 6.5%, *p* = 0.006), respectively (Additional Table 1).

**Fig. 1:**
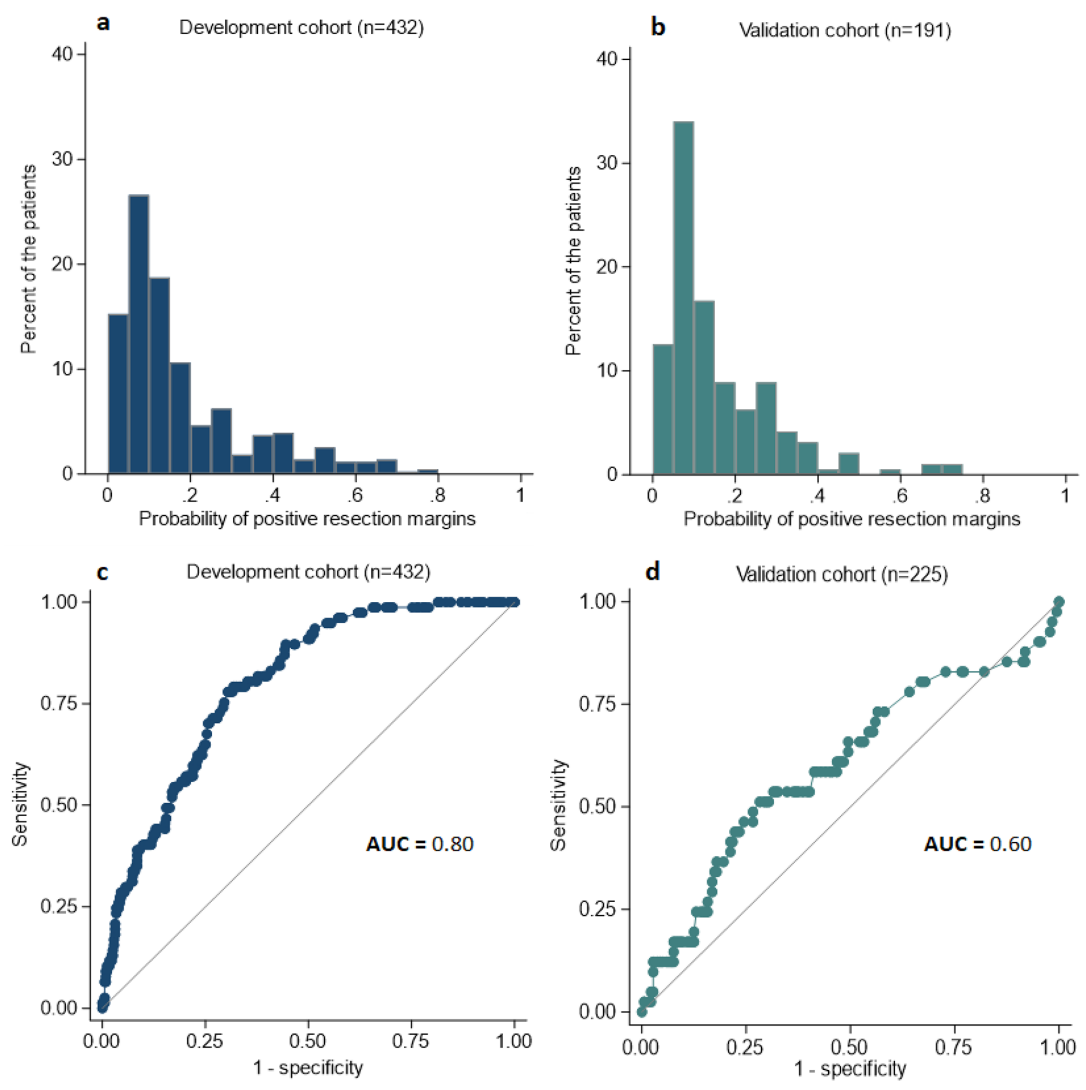
Predicted probabilities and receiver-operating characteristics curves

### External validation of the prediction model

The distribution patterns of the predicted probabilities of positive resection margins were relatively similar between the cohorts (Fig. 1a and b), although there was a higher proportion of patients in the validation cohort with a predicted 5–10% risk of positive resection margins, compared to the development cohort. The accuracy of the prediction model to discriminate between patients with positive margins and patients with clear resection margins after BCS in the development and in the validation cohorts, respectively, is illustrated by the ROC curves (Fig. 1c and d). The multivariable model predicted positive resection margins after BCS in the validation cohort with an AUC of 0.57 (95% CI: 0.45; 0.69) for the 191 patients with complete data and with a mean AUC of 0.60 (95% CI: 0.50–0.70) as in Fig. 1d over the ten imputed datasets of all the 225 patients (Additional Fig. 2), including the 34 cases in the validation cohort with missing values for distance to NAC (Table 1). The mean AUC of the extended model was not improved: 0.60 (95% CI: 0.48–0.71), when adding mammographic density as an additional preoperative variable to the prediction model.

**Fig. 2:**
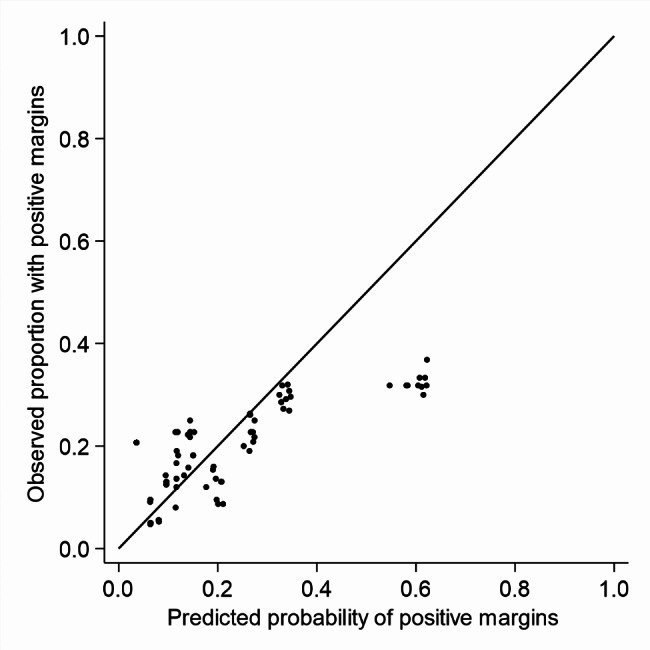
**Hosmer-Lemeshow calibration graph for the validation cohort**

Agreement between the predicted probabilities and the observed relative frequencies of positive margins is illustrated in the calibration curve of Hosmer-Lemeshow (Fig. 2) that was relatively good for 80% of the patients. For 10% of the patients with a low predicted risk of positive margins after BCS, the model underestimated the risk of positive resection margins. For the last 10% of the patients with the highest predicted risks of positive resection margins after BCS, the model overestimated the risk of positive margins after BCS. The underestimation of low risks and the overestimation of high risks lead to a mean calibration slope of 0.25 over the 10 imputed datasets and the mean calibration intercept was − 0.06 (Fig. 2).

## Discussion

External validation of a prediction model is important but rarely performed [29, 30].

We present external validation of a multivariable model predicting positive resection margins after BCS with an AUC of 0.60. One explanation for the low AUC is the differences in patient and tumor characteristics between the validation and development cohort. For example, DCIS, one of the strongest predictors in the original model, only 0.9% (2/225) of the patients in the validation cohort had DCIS vs. 11.1% (48/432) in the development cohort [10]. Another explanation for the lower AUC in the validation cohort is pure chance related to the small sample size of the validation cohort. The validation cohort had a lower fraction of patients with mammographic microcalcifications as compared to the development cohort due to the lower prevalence of DCIS and a higher percentage of patients with no visible tumor on preoperative mammography. This could be due to a higher proportion of patients with dense breasts in the validation cohort, although we do not have data on mammographic breast density from the development cohort, as this was not routinely reported at the Swedish site. In the development cohort more oncoplastic surgeries were performed, a technique which has shown to reduce the percentages of positive margins after BCS, due to larger volume of excised breast tissue [31, 32]. This could explain the lower number of positive margins in the development vs. the validation cohort, when calculated per number of invasive cancers.

The prediction model of Ellbrant et al. has previously been externally validated in a smaller sample size of 157 in situ and invasive breast cancer patients with an AUC of 0.75 [10]. The initial external validation cohort from Sweden had similar settings and patient demographics as in the original model, which may explain a higher AUC than that observed in the Danish validation cohort.

Two studies [20, 21] from different countries have previously performed external validation of a multivariable prediction model of positive margins after BCS by Pleijhuis et al. [14]. Ribeiro AL et al. [20] found a low performance of the model with an AUC of 0.51. Agostinho et al. [21] found no correlation between the predicting variables of the model and positive resection margins after BCS in their validation cohort. The poor external validity of these prediction models was mainly due to different settings and guidelines defining the outcome between the sites, indicating that external validation for prediction of positive resection margins is difficult to perform and site dependent.

We found that the additional available variable, high mammographic breast density in the validation cohort was associated with positive resection margins. This is in accordance with three previous studies that have shown an association between high mammographic breast density and positive resection margins [7, 8, 23], although other studies could not confirm that [12, 22, 33]. When adding mammographic breast density to the model, the AUC did not improve in the validation cohort, probably due to the low prevalence in the highest breast density category D.

The strength of this study is that at both sites, identical guidelines were used for defining a positive margin after BCS. Another strength is the use of multiple imputations of missing data to reduce the risk of selection bias, using as much of the validation dataset as possible.

In conclusion, the accuracy of the model to predict positive margins after BCS in the validation cohort was lower compared to the development cohort and did not further improve when mammographic breast density was included to the model, underscoring the difficulties in development of generalizable prediction models.

## Limitations

- Validation cohort: Low prevalence of the key predictors, DCIS in the core-needle biopsy and of oncoplastic surgeries.
- Several of the predictors had a distribution different from the development cohort or were retrospectively collected (distance to the NAC, microcalcifications and tumor size on mammography).
- Relatively small size of the validation cohort.

## Electronic supplementary material

Below is the link to the electronic supplementary material.

Supplementary Material 1

## Data Availability

All data of the included patients in the validation cohort were extracted from the patient’s medical file and captured in the Research Electronic Data Capture system. Data is available by contact to the corresponding author upon reasonable request.

## Abbreviations

BCS: Breast-conserving surgery
ROC: Receiver operating characteristic
AUC: Area under the curve
DCIS: Ductal carcinoma in situ
NACT: Neoadjuvant chemotherapy
MAR: Missing at random
NAC: Nipple-Areola complex
