# Supplementary material for: External validation of a multivariable prediction model for positive resection margins in breast-conserving surgery
Source: BMC Res Notes. 2025 Jan 27;18:36. doi: 10.1186/s13104-025-07103-8 (PMC11770974; doi:10.1186/s13104-025-07103-8)
Supplement: Supplementary file 1 — Supplementary Material 1 [file 13104_2025_7103_MOESM1_ESM.docx]

**Supplementary material**

**Additional Figure 1: Flow chart of study patients in the development and the validation cohort.**


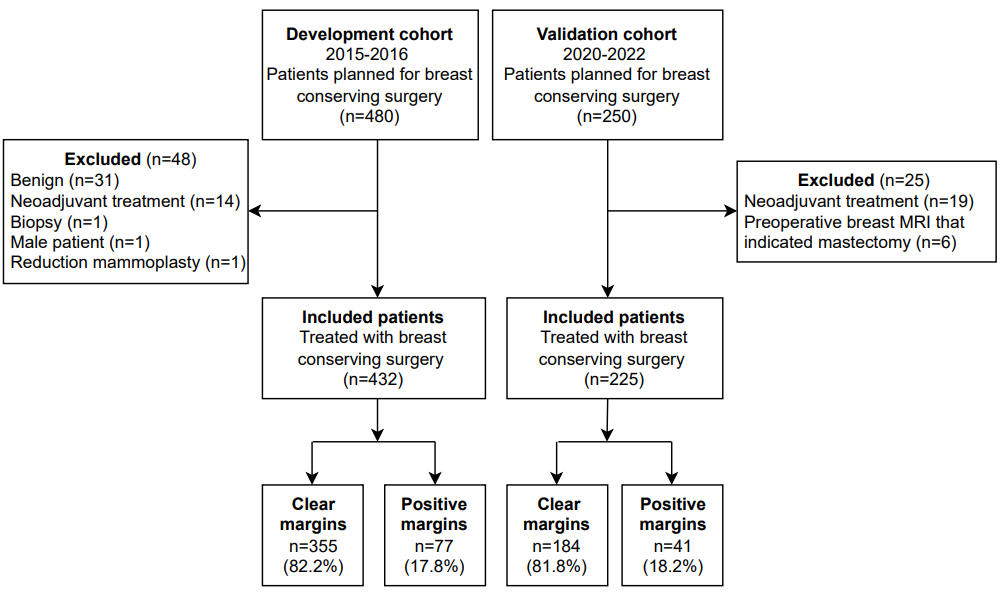


The patients in the development cohort, where included at Skåne University Hospital, Malmö, Sweden, and in the validation cohort at Aarhus University Hospital, Aarhus, Denmark.

**Additional Table 1: Patient characteristics of the development and validation cohort**

|  | **Development**  **Cohort (%)**  **n = 432** | **Validation**  **Cohort (%)**  **n = 225** | **P**  **Value** |
| --- | --- | --- | --- |
| **Demographic characteristics** |  |  |  |
| Age, years [median (min-max)]  < 50  50-59  60-69  ≥ 70 | 63 (28-90)  77 (17.8)  92 (21.3)  148 (34.3)  115 (26.6) | 65 (32-90)  13 (5.8)  51 (22.7)  93 (41.3)  68 (30.2) | **< 0.001^a^**  0.001^b^ |
| **Radiological features** |  |  |  |
| Visibility on mammography  Visible  Not visible | 404 (93.5)  28 (6.5) | 196 (87.1)  29 (12.9) | **0.006^c^** |
| Mammographic tumour size, mm  [median (min-max)]  ≤ 20 (T1)  21-50 (T2)  ˃ 50 (T3)  Not visible  Not measurable* | 14 (0-70)  338 (78.2)  61 (14.1)  5 (1.2)  28 (6.5)  0 (0) | 14 (4-77)  161 (71.6)  30 (13.3)  0 (0)  29 (12.9)  5 (2.2) | 0.588**^a^**  0.693^b^ |
| Mammographic calcifications  Yes  No | 115 (26.6)  317 (73.4) | 27 (12.0)  198 (88.0) | **< 0.001^c^** |
| Distance from NAC (cm)  < 5  ≥ 5  Missing** | 109 (25.2)  323 (74.8)  0 (0) | 45 (23.6)  146 (76.4)  34 (15.1) | 0.656**^c^** |
| Ultrasonographic tumour size, mm  [median (min-max)]  ≤ 20 (T1)  21-50 (T2)  ˃ 50 (T3)  Not visible  Unknown | 11 (0-70)  322 (80.1)  37 (9.2)  1 (0.2)  42 (10.4)  30 | 13 (3-40)  190 (84.4)  32 (14.2)  0 (0)  3 (1.33)  0 | **0.001^a^**  **0.048**^b^ |
| **Clinical-pathological findings** |  |  |  |
| Palpability  Palpable  Non-palpable | 227 (52.5)  205 (47.5) | 107 (47.6)  118 (52.4) | 0.225**^c^** |
| Tumour location  Superior medial quadrant  Superior lateral quadrant  Inferior lateral quadrant  Inferior medial quadrant  Retromammillary | 72 (16.7)  206 (47.7)  95 (22.0)  51 (11.8)  8 (1.9) | 41 (18.2)  134 (59.6)  23 (10.2)  15 (6.7)  12 (5.3) | **<0.001^c^** |
| Core-needle biopsy Lobular cancer  Yes  No | 49 (11.3)  383 (88.7) | 32 (14.2)  193 (85.8) | 0.287^c^ |
| Core-needle biopsy: DCIS  Yes  No | 48 (11.1)  384 (88.9) | 2 (0.9)  223 (99.1) | **< 0.001^c^** |
| **Type of surgery** |  |  |  |
| Partial mastectomy  Oncoplastic partial mastectomy | 309 (71.5)  123 (28.5) | 214 (95.1)  11 (4.9) | **< 0.001^c^** |

**Abbreviations:** NAC, Nipple-aerola-complex; DCIS, ductal carcinoma in situ. *In 5 cases,

the tumour was identified on mammography, but the tumour margins where not visible.

**Missing NAC due to no visible or no measurable tumour on mammography that was not

reported on ultrasound by the radiologist. a) two-sample t-test, b) linear trend test,

c) Pearson´s Chi-squared test.

**Additional Figure 2: The ROC curves for the ten imputations in the validation cohort (n=225).**
